# Detection of susceptibility loci on *APOA5* and *COLEC12* associated with metabolic syndrome using a genome-wide association study in a Taiwanese population

**DOI:** 10.18632/oncotarget.20967

**Published:** 2017-09-16

**Authors:** Eugene Lin, Po-Hsiu Kuo, Yu-Li Liu, Albert C. Yang, Shih-Jen Tsai

**Affiliations:** ^1^ Graduate Institute of Biomedical Sciences, China Medical University, Taichung, Taiwan; ^2^ Vita Genomics, Inc., Taipei, Taiwan; ^3^ TickleFish Systems Corporation, Seattle, WA, USA; ^4^ Department of Public Health, Institute of Epidemiology and Preventive Medicine, National Taiwan University, Taipei, Taiwan; ^5^ Center for Neuropsychiatric Research, National Health Research Institutes, Miaoli County, Taiwan; ^6^ Department of Psychiatry, Taipei Veterans General Hospital, Taipei, Taiwan; ^7^ Division of Psychiatry, National Yang-Ming University, Taipei, Taiwan; ^8^ Division of Interdisciplinary Medicine and Biotechnology, Beth Israel Deaconess Medical Center/Harvard Medical School, Boston, MA, USA

**Keywords:** gene-gene interactions, genome-wide association studies, metabolic syndrome, single nucleotide polymorphisms, Pathology Section

## Abstract

**Background:**

Although the association of single nucleotide polymorphisms (SNPs) with metabolic syndrome (MetS) has been reported in various populations in several genome-wide association studies (GWAS), the data is not conclusive. In this GWAS study, we assessed whether SNPs are associated with MetS and its individual components independently and/or through complex interactions in a Taiwanese population.

**Methods:**

A total of 10,300 Taiwanese subjects were assessed in this study. Metabolic traits such as waist circumference, triglyceride, high-density lipoprotein (HDL) cholesterol, systolic and diastolic blood pressure, and fasting glucose were measured.

**Results:**

Our data showed an association of MetS at the genome-wide significance level (*P* < 8.6 x 10^-8^) with two SNPs, including the rs662799 SNP in the apolipoprotein A5 (*APOA5*) gene and the rs16944558 SNP in the collectin subfamily member 12 (*COLEC12*) gene. Moreover, we identified the effect of *APOA5* rs662799 on triglyceride and HDL, the effect of rs1106475 in the actin filament associated protein 1 like 2 (*AFAP1L2*) gene on systolic blood pressure, and the effect of rs17667932 in the mediator complex subunit 30 (*MED30*) gene on fasting glucose. Additionally, we found that an interaction between the *APOA5* rs662799 and *COLEC12* rs16944558 SNPs influenced MetS, high triglyceride, and low HDL.

**Conclusions:**

Our study indicates that the *APOA5* and *COLEC12* genes may contribute to the risk of MetS and its individual components independently as well as through gene-gene interactions.

## INTRODUCTION

The metabolic syndrome (MetS), a chronic disease of metabolic dysregulation and a consequence of complicated interplay between genetic and environmental causes, is characterized by having large waist circumference plus two or more of the following physiologic abnormalities: raised triglyceride levels, low high-density lipoprotein (HDL) cholesterol levels, raised blood pressure, and raised glucose levels [1]. The manifestations of MetS encompass atherosclerosis, Alzheimer’s disease, cancer, cognitive disorders, coronary artery disease (CAD), hypertension, insulin resistance, lipid metabolism, obesity, and type 2 diabetes [2, 3]. Because of its escalating prevalence rate and an increased risk of chronic diseases such as CAD, MetS has become a leading public health concern in Taiwan and around the world as a result of excess energy intake and inactive lifestyles [2, 4]. In general, it has been estimated that at least one-quarter of the adult population has MetS worldwide [2]. We can certainly improve our long-term health by diagnosis MetS early with treatment [5]. Several lines of evidence support that MetS appears to be highly heritable [6]. In addition to making use of traditional candidate gene approaches, more and more genetic loci for MetS have been identified by leveraging hypothesis-free genome-wide association studies (GWAS) [6, 7]. It has long been recognized that genetic variants conferring susceptibility to MetS may diversify across ethnicities [6, 7].

Several GWAS have been conducted to identify susceptible genetic loci affecting MetS as an entity (Supplementary Table S1) [8-13]. A previous GWAS study by Kraja *et al.* indicated that there was a significant association of MetS with the apolipoprotein A5 (*APOA5*), BUD13 homolog (*BUD13*), cholesteryl ester transfer protein (*CETP*), lipoprotein lipase (*LPL*), and ZPR1 zinc finger (*ZPR1*) genes on data from 7 cohorts in Caucasian populations [8]. The following GWAS study by Kristiansson *et al.* reported that the *ZPR1* rs964184 SNP may contribute to the susceptibility for MetS in Finnish cohorts [9]. Moreover, another GWAS study by Jeong *et al.* implicated that two SNPs including rs11216126 and rs180349 may be involved with MetS susceptibility in a Korean population [10]. By using a GWAS analysis, Tekola-Ayele *et al.* also demonstrated that the rs73989312 SNP near the carbonic anhydrase 10 (*CA10*) gene and the rs77244975 SNP in the catenin alpha 3 (*CTNNA3*) gene may be a determinant of MetS in an African population [11]. Finally, a recent GWAS study by Zhu *et al.* showed that the *APOA5* rs651821 SNP and the rs671 SNP in the aldehyde dehydrogenase 2 family (*ALDH2*) gene are likely to influence MetS in Chinese subjects [12]. On the contrary, Zabaneh and Balding performed a GWAS study in a male Indian Asian population and detected no association of MetS with SNPs [13].

Based on a Taiwanese population, previous candidate-gene association studies showed a nominal association of MetS with several SNPs in the *APOA5*, *BUD13*, *CETP*, lipase A lysosomal acid type (*LIPA*), and five circadian clock genes including aryl hydrocarbon receptor nuclear translocator like (*ARNTL*), glycogen synthase kinase 3 beta (*GSK3B*), period circadian clock 3 (*PER3*), RAR related orphan receptor A (*RORA*), RAR related orphan receptor B (*RORB*); but none of these SNPs persisted significantly after performing Bonferroni correction [14, 15]. To our knowledge, no GWAS studies have been evaluated in a Taiwanese population. Given that the known genetic variations explain only a fraction of the heritability for MetS [9, 12], we hypothesized that potential genetic biomarkers of MetS susceptibility remain to be discovered. Furthermore, the interplay between genes in affecting MetS traits have not been fully evaluated in previous GWAS of other populations. In light of the aforementioned considerations, we thus searched for MetS susceptibility loci by performing a GWAS study with MetS per se as well as with its individual components in Taiwanese individuals. We also determined whether significant gene-gene interactions exist between some key genes in influencing MetS and individual components.

## RESULTS

Table 1 describes the demographic and clinical characteristics of the study population, including 1,811 MetS subjects and 8,489 non-MetS subjects. The MetS prevalence in our cohort was 17.6%. As shown in Table 1, there was a significant difference in waist circumference, triglyceride, HDL, blood pressure, and fasting glucose between the MetS and non-MetS subjects (all *P* < 0.0001, respectively).

**Table 1:** Demographic and clinical characteristics of study subjects.

Characteristic	Without MetS	With MetS	*P* value
No. of subjects (*n*)	8,489	1,811	
Age (years)	48.8±11.3	53.1±10.5	< 0.0001
Sex (male %)	47.6%	52.2%	0.0004
Waist circumference (cm)	82.0±9.0	94.1±8.3	< 0.0001
Triglyceride (mg/dl)	101.8±68.8	196.5±132.0	< 0.0001
HDL (mg/dl)	55.2±12.8	43.3±9.3	< 0.0001
Systolic blood pressure (mmHg)	114.8±16.5	129.4±17.2	< 0.0001
Diastolic blood pressure (mmHg)	71.4±10.5	79.4±11.2	< 0.0001
Fasting glucose (mg/dl)	93.6±16.4	110.7±34.1	< 0.0001

HDL = high-density lipoprotein cholesterol, MetS = metabolic syndrome.

Data are presented as mean ± standard deviation.

**Figure 1:**
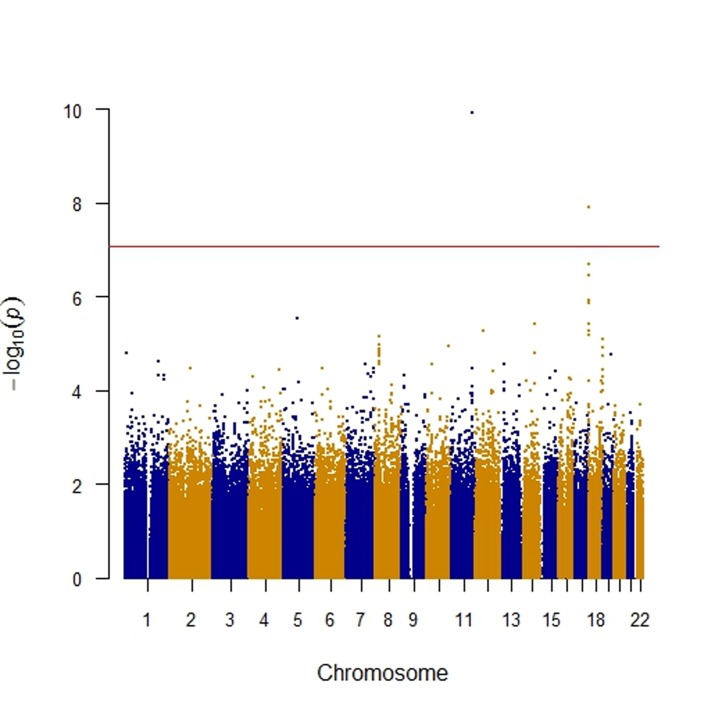
The Manhattan plot of genome-wide association of single nucleotide polymorphisms (SNPs) with the metabolic syndrome The Manhattan plot was constructed using the *P* values of SNPs, which was generated via logistic regression after adjustment for covariates including age and sex. The individual SNP is represented by a point, with higher points (higher negative log_10_ *P* values) indicating more significant association. The red horizontal line is the genome-wide significance level (*P* = 8.6 × 10^-8^), and points above the red horizontal line indicate SNPs with a P value of less than 8.6×10^−8^. Y-axis: -log_10_ (*P* value) of each SNP; X-axis: chromosomes labelled with blue and orange colors.

**Figure 2:**
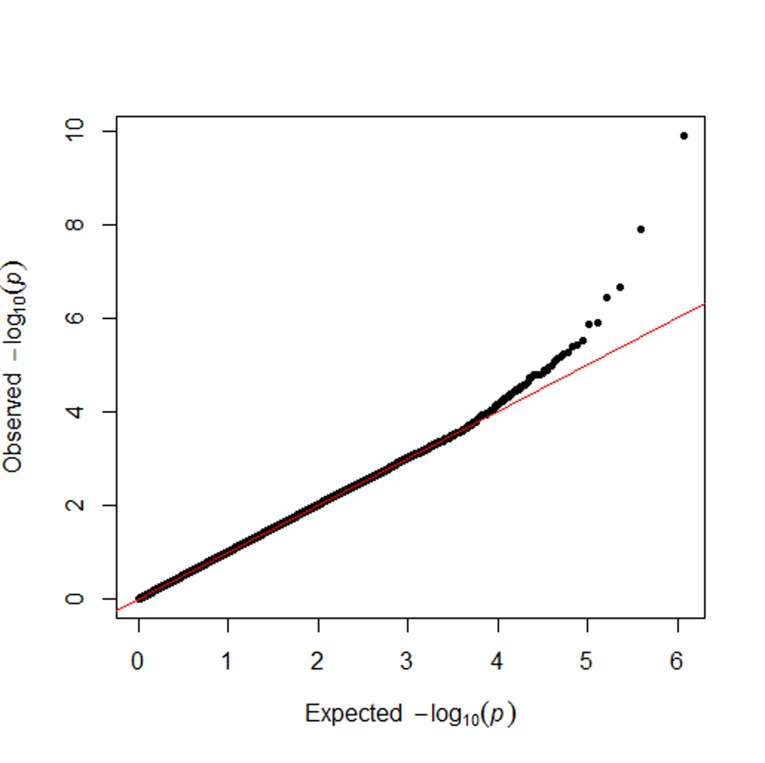
The QQ-plot of metabolic syndrome results The plots show observed and expected distributions of *P*-values from the genome-wide association study of the metabolic syndrome.

As shown in Table 2, we identified two key SNPs, including *APOA5* rs662799 and the rs16944558 SNP in the collectin subfamily member 12 (*COLEC12*) gene, associated with MetS per se at the genome-wide significance level (*P* < 8.6 x 10^-8^). As demonstrated in Table 2 for the *APOA5* rs662799 SNP, there was an indication of an increased MetS risk among the MetS and non-MetS subjects after adjustment of covariates such as age and sex for the dominant model (odds ratio (OR) = 1.40; 95% confidence interval (CI) = 1.27-1.56; *P* = 1.2 x10^-10^). Similarly, there was an indication of an increased risk of MetS among the subjects after adjustment of covariates for the dominant model in the *COLEC12* rs16944558 SNP (OR = 1.40; 95% CI = 1.25-1.57; *P* = 1.3 x10^-8^). The Manhattan and quantile-quantile (Q-Q) plots of GWAS for SNPs with MetS in terms of the dominant model is shown in Figures 1 and 2, respectively.

**Table 2:** Odds ratio analysis with odds ratios after adjustment for covariates between the MetS and two SNPs (including *APOA5* rs662799 and *COLEC12* rs16944558) with genome-wide significance.

Gene	SNP	Chr	A1	A2	Additive model			Dominant model			Recessive model		
					OR	95% CI	*P*	OR	95% CI	*P*	OR	95% CI	*P*
*APOA5*	rs662799	11	G	A	1.25	1.14-1.37	3.7 x10^-6^	1.40	1.27-1.56	**1.2 x10**^**-10**^	1.35	1.13-1.62	0.0012
*COLEC12*	rs16944558	18	T	C	1.20	1.12-1.30	1.2 x10^-6^	1.40	1.25-1.57	**1.3 x10**^**-8**^	1.18	1.04-1.34	0.0101

A1 = minor allele, A2 = major allele, Chr = chromosome, CI = confidence interval, MetS = metabolic syndrome, OR = odds ratio.

Analysis was obtained after adjustment for covariates including age and sex. *P* values of < 8.6 x 10^-8^ (genome-wide significance) are shown in bold.

Next, we examined the SNPs with MetS traits as quantitative measures, including waist circumference, triglyceride, HDL, systolic blood pressure, diastolic blood pressure, and fasting glucose. As shown in Table 3 and Supplementary Table S2, there was evidence of an association between *APOA5* rs662799 and triglyceride (additive model: *P* = 6.8 x10^-84^; dominant model: *P* = 7.2 x10^-52^; recessive model: *P* = 1.3 x10^-67^) at the genome-wide significance level. Moreover, *APOA5* rs662799 was associated with HDL (additive model: *P* = 2.8 x10^-26^; dominant model: *P* = 1.8 x10^-30^; recessive model: *P* = 5.7 x10^-18^) at the genome-wide significance level. Additionally, there was a significant difference in systolic blood pressure for the rs1106475 SNP in the actin filament associated protein 1 like 2 (*AFAP1L2*) gene (additive model: *P* = 7.68 x10^-8^) as well as in fasting glucose for the rs17667932 SNP in the mediator complex subunit 30 (*MED30*) gene (additive model: *P* = 2.27 x10^-14^; recessive model: *P* = 2.28 x10^-14^) among the subjects after adjustment of covariates at the genome-wide significance level (Table 3). Moreover, there was evidence of an association between rs17075725 in the glutamate metabotropic receptor 1 (*GRM1*) gene and waist circumference as well as between rs4893980 in the phosphodiesterase 11A (*PDE11A*) gene and diastolic blood pressure; however, this association did not reach genome-wide significance (Table 3). The Manhattan and Q-Q plots of GWAS for SNPs with MetS traits (including waist circumference, triglyceride, HDL, systolic blood pressure, diastolic blood pressure, and fasting glucose) in terms of the additive model are shown in Supplementary Figures 1 - 12, respectively.

**Table 3:** Linear regression models of associations between individual components of the MetS and top SNPs (that is, one SNP per gene with the smallest *P* value).

Gene	SNP	Chr	A1	A2	Additive model			Dominant model			Recessive model		
					BETA	SE	*P*	BETA	SE	*P*	BETA	SE	*P*
(a) Waist circumference													
*GRM1*	rs17075725	6	A	G	2.469	0.5125	1.48 x10^-6^	0.1784	0.2417	0.4606	4.946	1.024	1.39 x10^-6^
(b) Triglyceride													
*APOA5*	rs662799	11	G	A	32.91	1.68	**6.8 x10**^**-84**^	26.54	1.74	**7.2 x10**^**-52**^	57.51	3.29	**1.3 x10**^**-67**^
(c) HDL													
*APOA5*	rs662799	11	G	A	-2.47	0.23	**2.8 x10**^**-26**^	-2.75	0.24	**1.8 x10**^**-30**^	-3.92	0.45	**5.7 x10**^**-18**^
(d) Diastolic blood pressure													
*PDE11A*	rs4893980	2	T	C	1.228	0.2511	1.02 x10^-6^	0.2741	0.2095	0.1907	2.461	0.4967	7.34 x10^-7^
(e) Systolic blood pressure													
*AFAP1L2*	rs1106475	10	T	C	1.693	0.3148	**7.68 x10**^**-8**^	0.7763	0.3058	0.01115	3.245	0.6153	1.37 x10^-7^
(f) Fasting glucose													
*MED30*	rs17667932	8	C	T	30.29	3.962	**2.27 x10**^**-14**^	1.044	0.9626	0.278	60.57	7.923	**2.28 x10**^**-14**^

BETA = Beta coefficients, Chr = chromosome, HDL = high-density lipoprotein cholesterol, MetS = metabolic syndrome, SE = standard error.

Analysis was obtained after adjustment for covariates including age and sex. *P* values of < 8.6 x 10^-8^ (genome-wide significance) are shown in bold.

In addition, the generalized multifactor dimensionality reduction (GMDR) analysis was used to assess the impacts of combinations between two key SNPs (namely *APOA5* rs662799 and *COLEC12* rs16944558) in MetS and its individual components (as dichotomous measures) including age and sex as covariates. Table 4 summarizes the results obtained from GMDR analysis for two-way gene-gene interaction models in influencing MetS and its individual components with covariate adjustment. As shown in Table 4, there was a significant two-way model involving *APOA5* rs662799 and *COLEC12* rs16944558 (*P* < 0.001). The effects of these two-way models remained significant after Bonferroni correction (*P* < 0.05/6 = 0.008), indicating a potential gene-gene interaction between *APOA5* and *COLEC12* in influencing MetS. Moreover, there were two-way gene-gene interaction models in influencing individual components such as high triglyceride (*P* < 0.001) and low HDL (*P* < 0.001).

**Table 4:** Two-way gene-gene interaction models by using the GMDR method with adjustment for age and sex.

Phenotype	Two-way interaction model	Testing accuracy (%)	*P* value
MetS	*APOA5* rs662799, *COLEC12* rs16944558	53.98	**< 0.001**
High waist circumference^a^	*APOA5* rs662799, *COLEC12* rs16944558	50.34	0.335
High triglyceride^b^	*APOA5* rs662799, *COLEC12* rs16944558	58.72	**< 0.001**
Low HDL^c^	*APOA5* rs662799, *COLEC12* rs16944558	55.30	**< 0.001**
High blood pressure^d^	*APOA5* rs662799, *COLEC12* rs16944558	49.71	0.652
High fasting glucose^e^	*APOA5* rs662799, *COLEC12* rs16944558	50.15	0.461

GMDR = generalized multifactor dimensionality reduction, HDL = high-density lipoprotein cholesterol, MetS = metabolic syndrome.

P value was based on 1,000 permutations. Analysis was obtained after adjustment for covariates including age and sex.

P values of < 0.008 (Bonferroni correction: 0.05/6) are shown in bold.

^a^ Waist circumference ≥ 90 cm in male subjects, waist circumference ≥ 80 cm in female subjects.

^b^ Triglyceride ≥ 150 mg/dl.

^c^ HDL< 40 mg/dl in male subjects, HDL < 50 mg/dl in female subjects.

^d^ Systolic blood pressure ≥ 130 mmHg or diastolic blood pressure ≥ 85 mmHg.

^e^ Fasting glucose ≥ 100 mg/dl.

We further utilized multivariable logistic regression analysis with adjustment for age and sex to assess the two-way *APOA5* rs662799 and *COLEC12* rs16944558 interaction models selected by the GMDR method. As shown in Supplementary Table S3, the significant interactions were confirmed by logistic regression models (MetS: *P* = 2.3 x 10^-7^; high triglyceride: *P* = 2.0 x 10^-16^; low HDL: *P* = 2.3 x 10^-16^).

Finally, statistical power analysis revealed that the present study had a 99.9% power to detect associations of *APOA5* rs662799 and *COLEC12* rs16944558 with MetS among the MetS and non-MetS subjects.

## DISCUSSION

Our GWAS analysis is the first study to date to track down whether the main effects of SNPs are significantly associated with the risk of MetS and its individual components independently and/or through gene-gene interactions among Taiwanese individuals. In this study, we pinpointed that *APOA5* rs662799 and *COLEC12* rs16944558 were linked with MetS at the genome-wide significance level. Additionally, we identified *APOA5* rs662799 as a genome-wide significant locus for the individual components of MetS such as triglyceride and HDL. Our data also indicated that there was a genome-wide significant association of *AFAP1L2* rs1106475 with systolic blood pressure as well as a genome-wide significant association of *MED30* rs17667932 with fasting glucose. Finally, gene-gene interactions of *APOA5* and *COLEC12* may contribute to the risk of MetS, high triglyceride, and low HDL.

In the present study, we found that the *APOA5* rs662799 SNP may play an important role in the modulation of MetS in a Taiwanese population by using a GWAS study. In addition, we observed that there was a genome-wide significant association of *APOA5* rs662799 with triglyceride and HDL. The *APOA5* gene is located on chromosome 11q23 and encodes an apolipoprotein protein that has been indicated in regulating the plasma triglyceride levels, a major risk factor for CAD [14]. Although the *APOA5* rs662799 polymorphism has been widely implicated to affect the MetS risk, the relationship between the MetS and *APOA5* rs662799 has been ambiguous [14]. Our results are in agreement with those of several other studies [14, 16-22]. The *APOA5* rs662799 SNP has been reported to increase the risk of developing MetS in Caucasians [16], Japanese [17], Taiwanese [14, 18], Hong Kong [19], Chinese [20], and Korean [21] populations. However, some studies in Arabic [23], Caucasian [24-26], and Hispanic [27] populations have shown contrasting findings in comparison to the current study. Several meta-analysis studies have also suggested that the *APOA5* rs662799 SNP is associated with an elevated risk of acquiring MetS in Asians, but not in European populations [20, 22]. Moreover, Xu *et al.* performed a meta-analysis on data from 91 studies including 51,868 subjects in Asian, European, and other ethnic populations and detected a significant association of *APOA5* rs662799 with triglyceride and HDL [20]. This association also remained significant after the subgroup analysis stratified by the ethnicity including both Asian and European populations [20]. It should be pointed out that the G allele frequency of *APOA5* rs662799 differs noticeably between various ethnic populations, ranging from 27.58% in the present Taiwanese population, 7.14% in British subjects, 35.58% in Japanese subjects, 12.3% in African American subjects, to 25.73% in Han Chinese subjects as shown in public data from the 1000 Genomes Project (Supplementary Table S4).

In the present study, we found a genome-wide significant association of MetS with the *COLEC12* rs16944558 SNP in a Taiwanese population by using a GWAS study. The *COLEC12* gene is located on chromosome 18p11.32 and encodes a scavenger receptor protein, which is implicated in several functions linked with host defense [28]. It has been shown that the *COLEC12* gene plays a role in mediating the uptake of oxidized low density lipoprotein in vascular endothelial cells, indicating its association with lipid metabolism, one of the hallmarks of MetS [28, 29]. It has also been proposed that the *COLEC12* gene is involved in the clearance of amyloid beta, suggesting its contribution to Alzheimer’s disease [30]. Furthermore, MetS has been correlated with age-related mental disorders such as Alzheimer’s disease in previous studies [31, 32]. Thus, we speculate that *COLEC12* may contribute to susceptibility to MetS because MetS is in turn associated with lipid metabolism and Alzheimer’s disease. It should be noted that the T allele frequency of *COLEC12* rs16944558 varies considerably between different ethnic populations, ranging from 44.16% in the present Taiwanese population, 9.34% in British subjects, 39.42% in Japanese subjects, 22.95% in African American subjects, to 44.66% in Han Chinese subjects (Supplementary Table S4).

In this study, another finding was a positive association of *AFAP1L2* rs1106475 with systolic blood pressure at the genome-wide significance level. The *AFAP1L2* gene, also known as *XB130*, is located on chromosome 10q25.3 and encodes an adaptor protein that can affect downstream proteins in signaling pathways even though the adaptor protein lacks enzyme catalytic activity [33, 34]. Previous studies have shown that the *AFAP1L2* gene is involved in gene regulation, cell growth, cell migration, cell survival, and cell invasion via the cAMP-cSrc-phosphoinositide 3-kinase/Akt pathway [33, 34]. The *AFAP1L2* gene was also found to be associated with tumorigenesis in multiple studies [35, 36]. It is of note that the *AFAP1L2* gene has been proposed to interact with the telomeric repeat binding factor 1 (*TERF1*) gene, which mediates telomerase and telomere functions [37]. Moreover, it has previously been reported that telomere length was associated with systolic blood pressure and hypertension [38, 39]. Thus, we speculate that *AFAP1L2* may contribute to susceptibility to systolic blood pressure because the gene-gene interaction between *AFAP1L2* and *TERF1* may in turn influence systolic blood pressure.

On another note, our analysis indicated that there was a genome-wide significant association of *MED30* rs17667932 with fasting glucose. The *MED30* gene is located on chromosome 8q24.11 and encodes one of the subunits of the Mediator complex, which is involved in various processes for transcription such as the organization of chromatin architecture [40]. Studies have shown that the Mediator complex is associated with various diseases such as glucose metabolism, lipid metabolism, cardiovascular disease (CVD), metabolic disorders, and neurological disorders [41, 42]. In an animal study, Krebs et al. reported that a mutation in the *MED30* gene results in lethal cardiomyopathy in the mouse heart, indicating a potential role of *MED30* in human CVD-related metabolic disorders [43].

By using the GMDR approach, we further inferred the epistatic effects between *APOA5* and *COLEC12* in influencing MetS and its individual components such as high triglyceride and low HDL. To our knowledge, no other study has been conducted to evaluate gene-gene interactions between these two genes. Besides the statistical significance, the potential biological mechanism under the interaction models was our concern. The functional relevance of the interactive effects of *APOA5* and *COLEC12* on MetS, high triglyceride, and low HDL remains to be elucidated. It was further speculated that the *APOA5* and *COLEC12* genes may be involved in the same pathways or pathology. It has been reported that overexpression of human *APOA5* in mice is correlated with decreased plasma triglyceride levels in an animal study [44]. Likewise, it has been shown that *APOA5* rs662799 is involved in the regulation of gene transcription due to its location in the promoter region and thereby considerably impact serum apolipoprotein A5 levels [17]. Moreover, both *APOA5* and *COLEC12* are known to play a key role in lipid metabolism [8, 28, 29].

This study has both strengths and limitations. The main weakness is that the results regarding *APOA5* did not represent novel findings although they were confirmed by other independent populations. On the other hand, the other novel findings relevant to *COLEC12*, *AFAP1L2*, and *MED30* were not verified by other independent populations. Thus, our observations require much more research to assess if the findings are replicated in diversified ethnic populations [45, 46]. In future work, prospective clinical trials with other ethnic populations are necessary to facilitate a thorough evaluation of the association and interactions of the investigated SNPs with MetS and its individual components [47-49]. By contrast, a major strength of our study is that we employed rigorously phenotyped MetS cases and healthy controls without MetS from the Taiwan Biobank to assess gene-gene interactions between the investigated genes.

## CONCLUSIONS

In conclusion, we carried out a GWAS analysis of the association as well as gene-gene interactions with MetS and its individual components in Taiwanese subjects. Our findings demonstrate that the *APOA5* and *COLEC12* genes may affect the prevalence of MetS independently and through complex gene-gene interactions. Furthermore, the *APOA5*, *AFAP1L2*, and *MED30* genes are a determinant of MetS component factors such as triglyceride, HDL, systolic blood pressure, and fasting glucose. Independent replication studies with larger sample sizes is essential to provide further insights into the role of these genes investigated in this study.

## MATERIALS AND METHODS

### Study population

This study included Taiwanese subjects from the Taiwan Biobank [14, 15, 32, 50-55]. This biobank collected specimens and associated data from the general Taiwanese population with no history of cancer through recruitment centers across Taiwan during 2013-2015 [14, 15, 32, 50-55]. This biobank is mainly funded by the Taiwanese government and aims to provide researchers with opportunities for collaboration to facilitate public health-related research concerning local common chronic diseases [50]. The study cohort consisted of 10,300 participants. Individuals who could perform activities of daily living, were aged 30-70 years, and were self-reported as being of Taiwanese Han Chinese ancestry were included in this study [51]. Individuals with a history of cancer or nonresidents of Taiwan were excluded [51]. Ethical approval for the study was granted by the Institutional Review Board of the Taiwan Biobank before conducting the study. Each subject signed the approved informed consent form. All experiments were performed in accordance with relevant guidelines and regulations.

### Metabolic syndrome

Measurements of metabolic traits (including as waist circumference, triglyceride, high-density lipoprotein cholesterol, systolic and diastolic blood pressure, and fasting glucose) were obtained when participants underwent general health examinations [14, 50, 51]. To assess MetS, we used the International Diabetes Federation definition, which requires that the participant represented by central obesity (defined as waist circumference ≥ 90 cm in male subjects and ≥ 80 cm in female subjects) plus the presence of two or more of the following four components: (1) triglycerides ≥ 150 mg/dl; (2) HDL cholesterol < 40 mg/dl in male subjects and < 50 mg/dl in female subjects; (3) systolic blood pressure ≥ 130 mmHg or diastolic blood pressure ≥ 85 mmHg; and (4) fasting plasma glucose ≥ 100 mg/dl [56]. Two measurements of blood pressure were taken in both arms at least 10-15 minutes apart in the sitting position. These measurements were averaged to obtain the final blood pressure used in this study.

### Genotyping

DNA was isolated from blood samples using a QIAamp DNA blood kit following the manufacturer’s instructions (Qiagen, Valencia, CA, USA). The quality of the isolated genomic DNA was evaluated using agarose gel electrophoresis, and the quantity was determined by spectrophotometry [57]. SNP genotyping was carried out using the custom Taiwan BioBank chips and run on the Axiom Genome-Wide Array Plate System (Affymetrix, Santa Clara, CA, USA). To efficiently obtain maximal genetic information from Taiwanese Han Chinese samples, the custom Taiwan BioBank chips were designed by using SNPs on the Axiom Genome-Wide CHB 1 Array (Affymetrix, Inc., Santa Clara, CA, USA) with minor allele frequencies (MAFs) ≥ 5%, by using SNPs in exons with MAFs > 10% on the Human Exome BeadChip (Illumina, Inc., San Diego, CA, USA), and by using SNPs previously reported in ancestry information panels, cancer studies, and pharmacogenetic studies [51]. To appraise the performance of the custom Taiwan BioBank chip, 70 unrelated Taiwanese individuals were genotyped by using both the custom Taiwan BioBank chip and the Axiom Genome-Wide CHB 1 Array and a high average concordance rate of 99.55% was obtained for the SNPs in the 70 subjects [51].

In this study, quality criteria for SNP exclusion from further analyses were the following: due to failure to achieve Hardy-Weinberg equilibrium (P < 1 x 10^-6^), due to a genotyping call rate < 90%, or due to MAF < 1%. After the quality control procedure, a total of 583,469 SNPs were used as the basis for SNP function prediction.

### Statistical analysis

Categorical data were evaluated using the chi-square test. We conducted the Student’s t-test to compare the difference in the means from two continuous variables. To estimate the association of the investigated SNP with MetS, we conducted a logistic regression analysis to evaluate the ORs and their 95% CIs, adjusting for covariates including age and sex [58]. Furthermore, we estimated the association of the investigated SNP with individual components of MetS by using logistic regression analysis, adjusting for age and sex [59]. The genotype frequencies were assessed for Hardy-Weinberg equilibrium using a χ^2^ goodness-of-fit test with 1 degree of freedom (i.e. the number of genotypes minus the number of alleles). Multiple testing was adjusted by the Bonferroni correction. The criterion for significance was set at P < 0.05 for all tests. Data are presented as the mean ± standard deviation.

To investigate gene-gene interactions, we employed the GMDR method [60]. We tested two-way interactions using 10-fold cross-validation. The GMDR software provides some output parameters, including the testing accuracy and empirical P values, to assess each selected interaction. Moreover, we provided age and sex as covariates for gene-gene interaction models in our interaction analyses. Permutation testing obtains empirical P values of prediction accuracy as a benchmark based on 1,000 shuffles. In order to correct for multiple testing, we applied a conservative Bonferroni correction factor for the number of tests employed in the GMDR analysis.

Based on the effect sizes in this study, the power to detect significant associations was evaluated by QUANTO software (http://biostats.usc.edu/Quanto.html). The Manhattan and Q-Q plots were drawn by using the R package ‘qqman’.

## SUPPLEMENTARY MATERIALS FIGURES AND TABLES




