# Supplementary material for: Detection of susceptibility loci on APOA5 and COLEC12 associated with metabolic syndrome using a genome-wide association study in a Taiwanese population
Source: Oncotarget. 2017 Sep 16;8(55):93349–59. doi: 10.18632/oncotarget.20967 (PMC5706800; doi:10.18632/oncotarget.20967)
Supplement: Supplementary file 1 [file oncotarget-08-93349-s001.pdf]

Detection of susceptibility loci on *APOA5* and *COLEC12* associated with metabolic syndrome using a genome-wide association study in a Taiwanese population

SUPPLEMENTARY MATERIALS

For Supplementary Tables see in Supplementary Files.

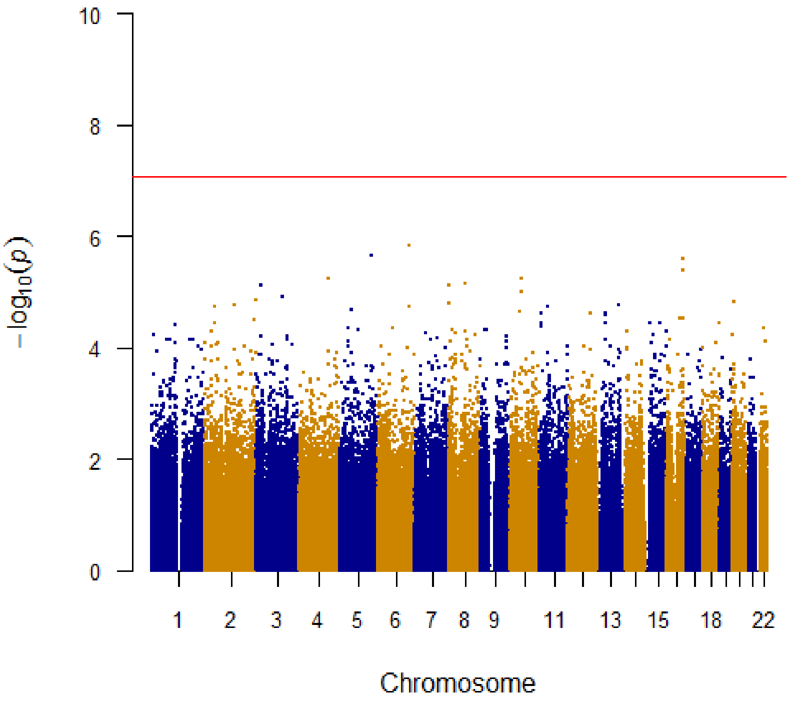

Supplementary Figure 1: The Manhattan plot of waist circumference results. The Manhattan plot was constructed using the P values of SNPs with waist circumference in terms of the additive model, which was generated via linear regression after adjustment for covariates including age and sex.

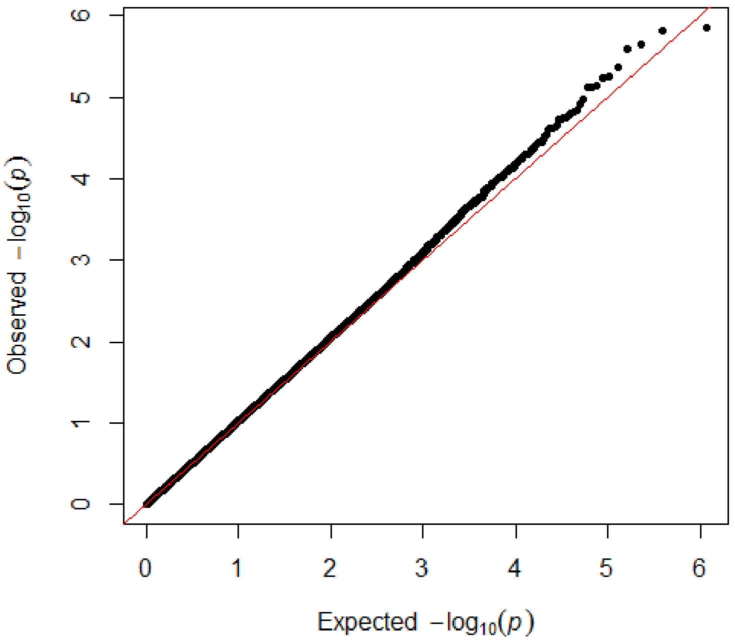

Supplementary Figure 2: The QQ-plot of waist circumference results. The plots show observed and expected distributions of P-values from the genome-wide association study of waist circumference.

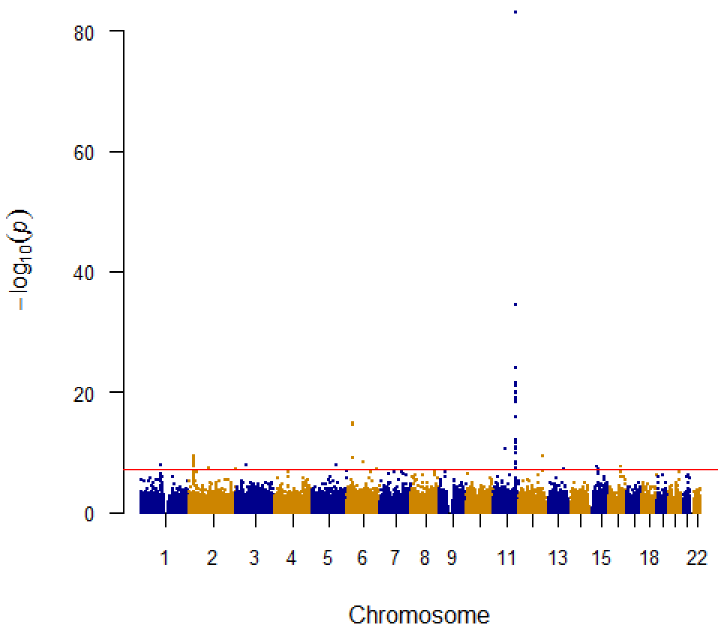

Supplementary Figure 3: The Manhattan plot of triglyceride results. The Manhattan plot was constructed using the P values of SNPs with triglyceride in terms of the additive model, which was generated via linear regression after adjustment for covariates including age and sex.

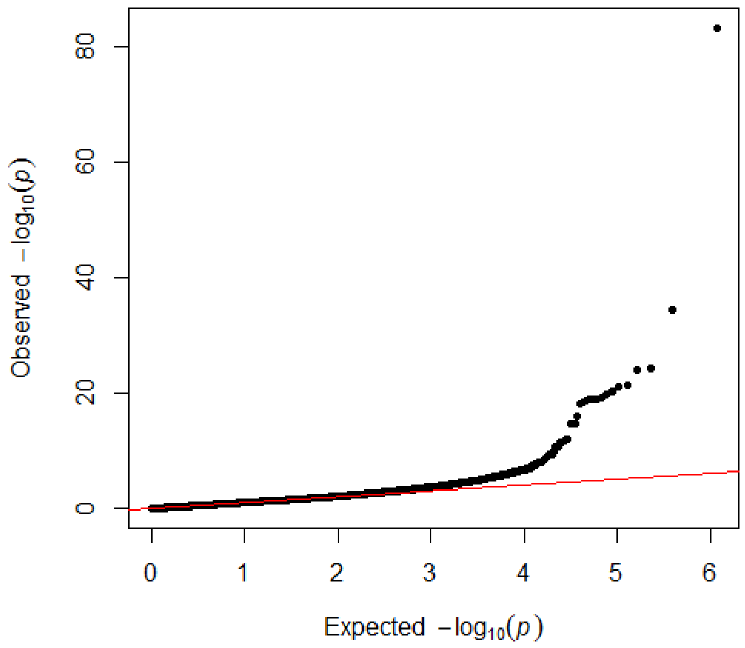

Supplementary Figure 4: The QQ-plot of triglyceride results. The plots show observed and expected distributions of P-values from the genome-wide association study of triglyceride.

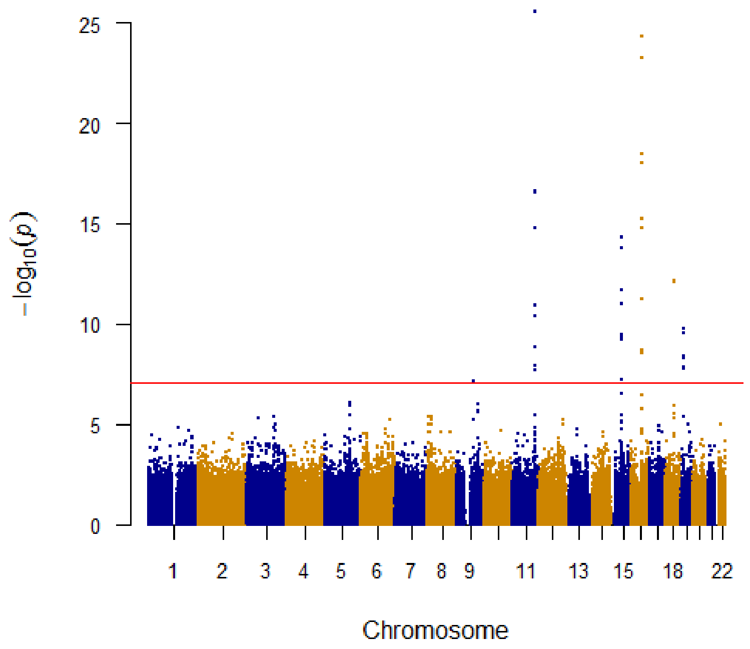

Supplementary Figure 5: The Manhattan plot of high-density lipoprotein (HDL) cholesterol results. The Manhattan plot was constructed using the P values of SNPs with HDL cholesterol in terms of the additive model, which was generated via linear regression after adjustment for covariates including age and sex.

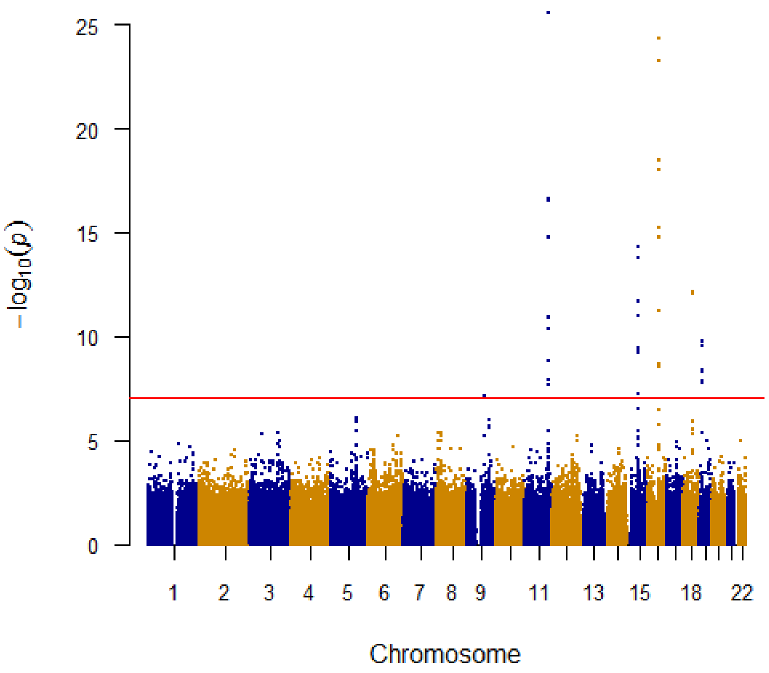

Supplementary Figure 5: The Manhattan plot of high-density lipoprotein (HDL) cholesterol results. The Manhattan plot was constructed using the P values of SNPs with HDL cholesterol in terms of the additive model, which was generated via linear regression after adjustment for covariates including age and sex.

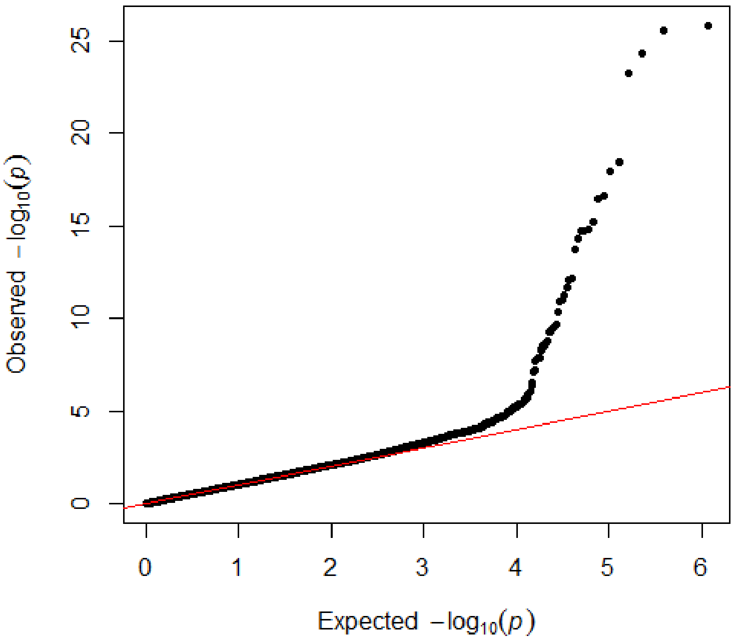

Supplementary Figure 6: The QQ-plot of high-density lipoprotein (HDL) cholesterol results. The plots show observed and expected distributions of P-values from the genome-wide association study of HDL cholesterol.

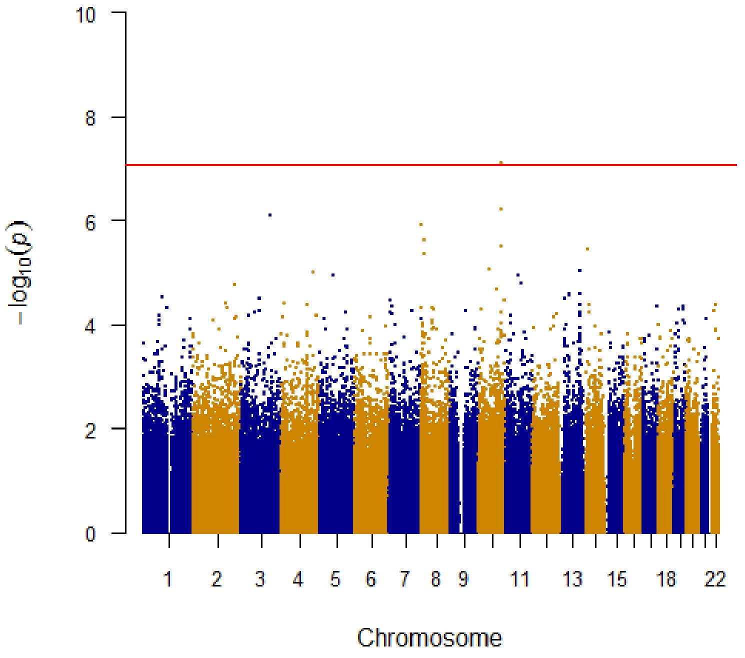

Supplementary Figure 7: The Manhattan plot of systolic blood pressure results. The Manhattan plot was constructed using the P values of SNPs with systolic blood pressure in terms of the additive model, which was generated via linear regression after adjustment for covariates including age and sex.

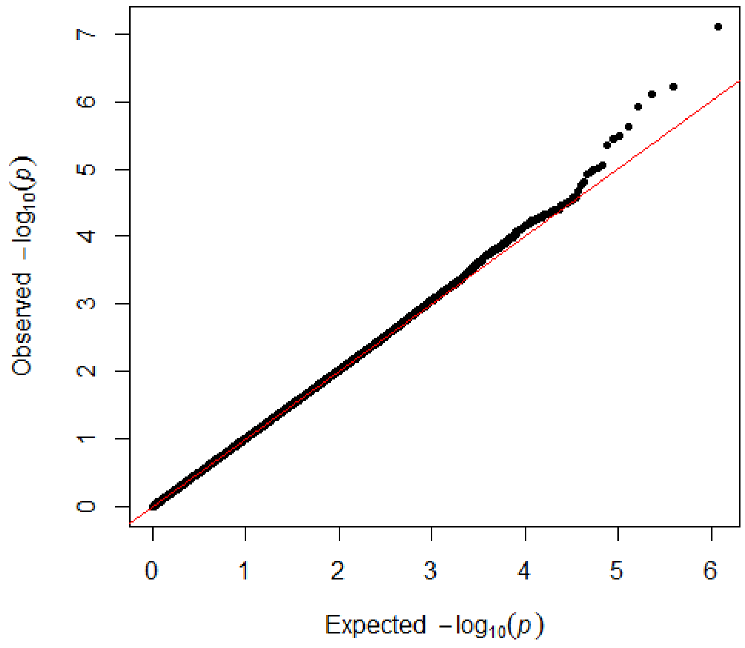

Supplementary Figure 8: The QQ-plot of systolic blood pressure results. The plots show observed and expected distributions of P-values from the genome-wide association study of systolic blood pressure.

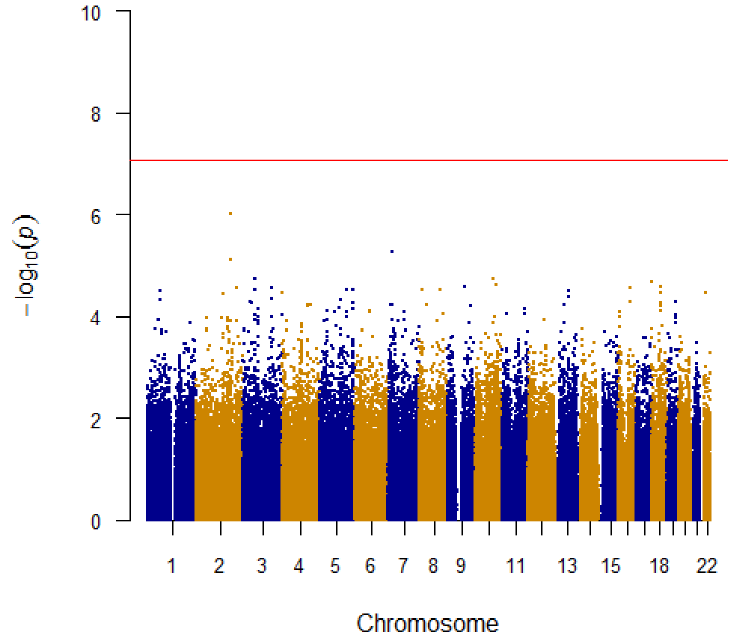

Supplementary Figure 9: The Manhattan plot of diastolic blood pressure results. The Manhattan plot was constructed using the P values of SNPs with diastolic blood pressure in terms of the additive model, which was generated via linear regression after adjustment for covariates including age and sex.

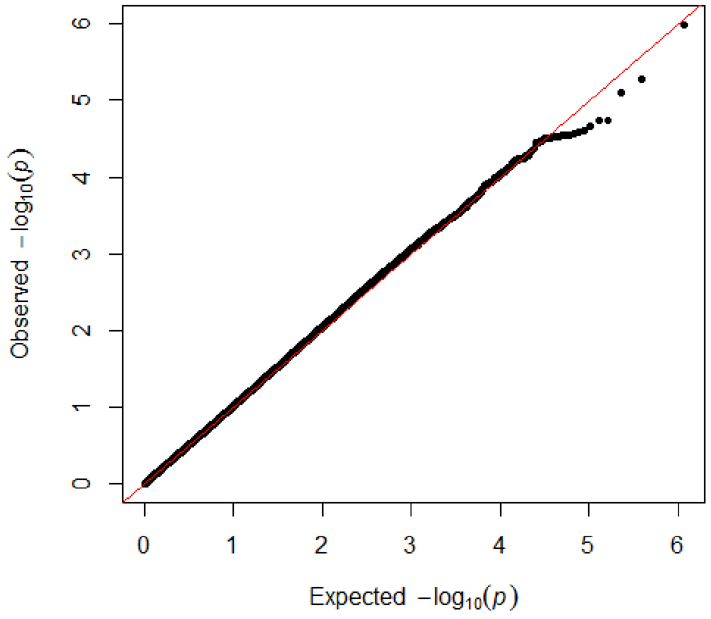

Supplementary Figure 10: The QQ-plot of diastolic blood pressure results. The plots show observed and expected distributions of P-values from the genome-wide association study of diastolic blood pressure.

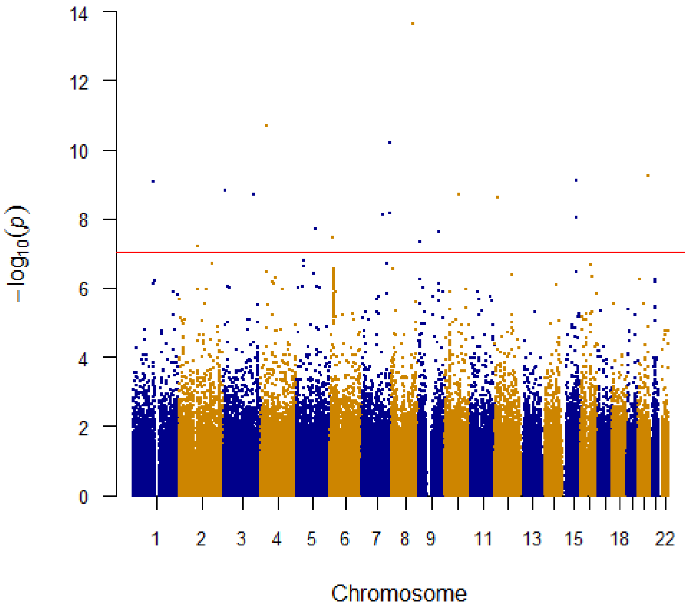

Supplementary Figure 11: The Manhattan plot of fasting glucose results. The Manhattan plot was constructed using the P values of SNPs with fasting glucose in terms of the additive model, which was generated via linear regression after adjustment for covariates including age and sex.

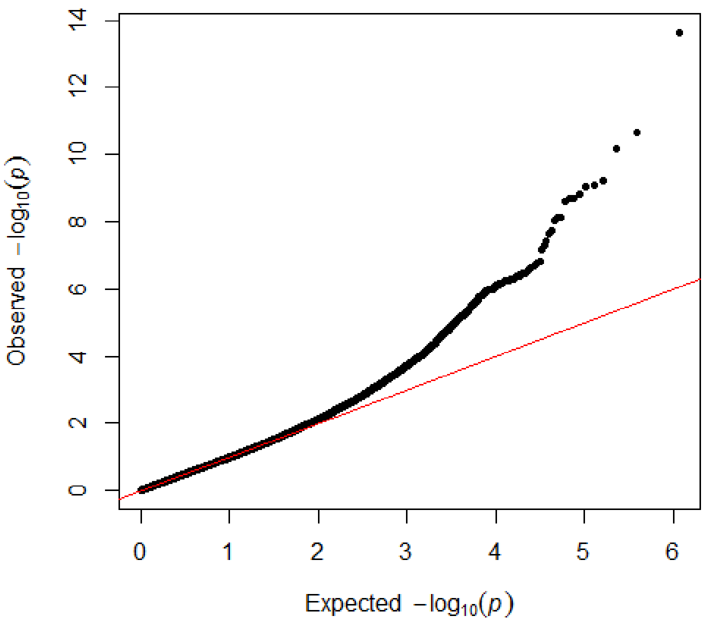

Supplementary Figure 12: The QQ-plot of fasting glucose results. The plots show observed and expected distributions of P-values from the genome-wide association study of fasting glucose.
