# Supplementary material for: Detection of susceptibility loci on APOA5 and COLEC12 associated with metabolic syndrome using a genome-wide association study in a Taiwanese population
Source: Oncotarget. 2017 Sep 16;8(55):93349–59. doi: 10.18632/oncotarget.20967 (PMC5706800; doi:10.18632/oncotarget.20967)
Supplement: Supplementary file 2 [file oncotarget-08-93349-s002.doc]

**Supplementary Table 1.** Genome-wide association studies with MetS among different ethnic populations.

| **Study** | **Population** | **No. of participants** | **Results** |
| --- | --- | --- | --- |
| Zabaneh and Balding 2010 | male Indian Asian | 4,560 | No association with MetS |
| Kraja *et al.* 2011 | Caucasian | 22,161 | Significant association of MetS with *APOA5*, *BUD13*, *CETP*, *LPL*, and *ZPR1* |
| Kristiansson *et al.* 2012 | Finnish | 10,564 | Significant association of MetS with the *ZPR1* rs964184 SNP |
| Jeong *et al.* 2014 | Korean | 8,574 | Significant association of MetS with the rs11216126 and rs180349 SNPs |
| Tekola-Ayele *et al.* 2015 | African | 1,427 | Significant association of MetS with the *CA10* rs73989312 and *CTNNA3* rs77244975 SNPs |
| Zhu *et al.* 2017 | Chinese | 10,978 | Significant association of MetS with the *APOA5* rs651821 and *ALDH2* rs671 SNPs |

*ALDH2* = aldehyde dehydrogenase 2 family, *APOA5* = apolipoprotein A5, *BUD13* = BUD13 homolog, *CA10* = carbonic anhydrase 10, *CETP*= cholesteryl ester transfer protein, *CTNNA3* = catenin alpha 3, *LPL* = lipoprotein lipase, MetS = metabolic syndrome, SNP = single nucleotide polymorphism, *ZPR1*= ZPR1 zinc finger.

**Supplementary Table 2.** Linear regression models of associations between individual components of the MetS and two key SNPs in the *APOA5* and *COLEC12* genes (including *APOA5* rs662799 and *COLEC12* rs16944558).

| **Individual components**  **of the MetS** | **Additive model** | | | **Dominant model** | | | **Recessive model** | | |
| --- | --- | --- | --- | --- | --- | --- | --- | --- | --- |
|  | **BETA** | **SE** | **P** | **BETA** | **SE** | **P** | **BETA** | **SE** | **P** |
| (1) *APOA5* rs662799 | | | | | | | | | |
| Waist circumference | -0.29 | 0.18 | 0.1048 | 0.04 | 0.18 | 0.8362 | -0.65 | 0.35 | 0.0629 |
| Triglyceride | 32.91 | 1.68 | **6.8 x10^-84^** | 26.54 | 1.74 | **7.2 x10^-52^** | 57.51 | 3.29 | **1.3 x10^-67^** |
| HDL | -2.47 | 0.23 | **2.9 x10^-26^** | -2.75 | 0.24 | **1.8 x10^-30^** | -3.92 | 0.45 | **5.7 x10^-18^** |
| Diastolic blood pressure | -0.05 | 0.20 | 0.7997 | 0.09 | 0.20 | 0.6511 | -0.16 | 0.38 | 0.6853 |
| Systolic blood pressure | 0.22 | 0.30 | 0.4565 | 0.30 | 0.31 | 0.3222 | 0.32 | 0.58 | 0.5763 |
| Fasting glucose | 0.50 | 0.40 | 0.2166 | 0.09 | 0.42 | 0.8311 | 1.03 | 0.79 | 0.1881 |
| (2) *COLEC12* rs16944558 | | | | | | | | | |
| Waist circumference | 0.41 | 0.13 | 0.0020 | 0.65 | 0.20 | 0.0011 | 0.46 | 0.23 | 0.0457 |
| Triglyceride | 3.04 | 1.27 | 0.0166 | 5.20 | 1.90 | 0.0061 | 3.11 | 2.22 | 0.1613 |
| HDL | -0.39 | 0.17 | 0.0234 | -0.76 | 0.26 | 0.0032 | -0.32 | 0.30 | 0.2878 |
| Diastolic blood pressure | 0.14 | 0.15 | 0.3261 | 0.44 | 0.22 | 0.0441 | -0.02 | 0.25 | 0.9387 |
| Systolic blood pressure | 0.35 | 0.22 | 0.1141 | 0.59 | 0.33 | 0.0743 | 0.36 | 0.38 | 0.3468 |
| Fasting glucose | 0.64 | 0.30 | 0.0314 | 0.84 | 0.45 | 0.0620 | 0.89 | 0.52 | 0.0902 |

BETA = Beta coefficients, HDL = high-density lipoprotein cholesterol, MetS = metabolic syndrome, SE = standard error.

Analysis was obtained after adjustment for covariates including age and gender.

P values of < 8.6 x 10^-8^ (genome-wide significance) are shown in bold.

**Supplementary Table 3.** Multivariable logistic regression analysis for the *APOA5* rs662799 and *COLEC12* rs16944558 interaction model.

| **Two-way interaction model** | **OR** | **95% CI** | **P value^b^** |
| --- | --- | --- | --- |
| (a) MetS | | | |
| *APOA5* rs662799 (AA genotype) with  *COLEC12* rs16944558 (TT+TC genotype)^a^ | 1 |  |  |
| *APOA5* rs662799 (AA genotype) with  *COLEC12* rs16944558 (CC genotype) | 0.69 | 0.58-0.82 | **2.1 x 10^-5^** |
| *APOA5* rs662799 (GG genotype) with  *COLEC12* rs16944558 (CC genotype) | 1.01 | 0.86-1.19 | 0.8750 |
| *APOA5* rs662799 (GG+GA genotype) with  *COLEC12* rs16944558 (TT+TC genotype) | 1.38 | 1.22-1.55 | **2.3 x 10^-7^** |
| (b) High triglyceride^c^ | | | |
| *APOA5* rs662799 (AA genotype) with  *COLEC12* rs16944558 (TT+TC genotype)^a^ | 1 |  |  |
| *APOA5* rs662799 (AA genotype) with  *COLEC12* rs16944558 (CC genotype) | 0.77 | 0.66-0.91 | **0.0023** |
| *APOA5* rs662799 (GG genotype) with  *COLEC12* rs16944558 (CC genotype) | 1.64 | 1.42-1.90 | **2.9 x 10^-11^** |
| *APOA5* rs662799 (GG+GA genotype) with  *COLEC12* rs16944558 (TT+TC genotype) | 2.01 | 1.79-2.26 | **2.0 x 10^-16^** |
| (c) Low HDL^d^ | | | |
| *APOA5* rs662799 (AA genotype) with  *COLEC12* rs16944558 (TT+TC genotype)^a^ | 1 |  |  |
| *APOA5* rs662799 (AA genotype) with  *COLEC12* rs16944558 (CC genotype) | 0.81 | 0.70-0.94 | **0.0052** |
| *APOA5* rs662799 (GG genotype) with  *COLEC12* rs16944558 (CC genotype) | 1.32 | 1.15-1.52 | **6.0 x 10^-5^** |
| *APOA5* rs662799 (GG+GA genotype) with  *COLEC12* rs16944558 (TT+TC genotype) | 1.57 | 1.41-1.74 | **2.3 x 10^-16^** |

CI = confidence interval, HDL = high-density lipoprotein cholesterol, MetS = metabolic syndrome, OR = odds ratio.

^a^ Reference.

^b^ Versus reference. Analysis was obtained after adjustment for covariates including age and gender. P values of < 0.05 are shown in bold.

^c^ Triglyceride ≥ 150 mg/dl.

^d^ HDL< 40 mg/dl in male subjects, HDL < 50 mg/dl in female subjects.

**Supplementary Table 4.** MAF in various ethnic populations for four key SNPs identified in our GWAS study.

| **Gene** | **CHR** | **SNP** | **A1** | **A2** | **MAF** | | | | |  |
| --- | --- | --- | --- | --- | --- | --- | --- | --- | --- | --- |
|  |  |  |  |  | **Taiwanese**^a^ | **British**^b^ | **Japanese**^b^ | **African Americans**^b^ | **Han Chinese**^b^ | |
| *AFAP1L2* | 10 | rs1106475 | T | C | 0.2605 | 0.1484 | 0.2115 | 0.0410 | 0.1942 | |
| *APOA5* | 11 | rs662799 | G | A | 0.2758 | 0.0714 | 0.3558 | 0.1230 | 0.2573 | |
| *COLEC12* | 18 | rs16944558 | T | C | 0.4416 | 0.0934 | 0.3942 | 0.2295 | 0.4466 | |
| *MED30* | 8 | rs17667932 | C | T | 0.0247 | 0.0934 | 0.1154 | 0.0328 | 0.0243 | |

A1 = minor allele, A2 = major allele, Chr = chromosome, MAF = minor allele frequency.

^a^ Data from the present study.

^b^ Data from the 1000 Genomes Project ([http://www.1000genomes.org](http://www.1000genomes.org/)).
